# Ship Fever

**Published:** 1849-02

**Authors:** Elizabeth Blackwell


### BUFFALO MEDICAL JOURNAL

AND

### MONTHLY REVIEW.

VOL. 4. FEBRUARY, 1849. NO. 9.

### ORIGINAL COMMUNICATIONS.

ART. I.—*Ship Fever. An Inaugural Thesis, submitted for the degree of M. D., at Geneva Medical College, Jan.* 1849. By Elizabeth Blackwell, M. D.

The summer of 1847 was distinguished by the epidemic brought to our seaport towns by means of the crowded emigrant ships, which arrived in great numbers from Europe, Our hospitals were filled to overflowing with patients in every stage of the disease,—many died in the receiving wards, many more only entered the hospital wards to prove the inefficiency of the resources of medical art. An examination of the nature of this disease, showed it to be a species of Typhus.

Much discussion has arisen in the Profession, in relation to the distinction between typhus and typhoid fever. The majority of French writers, and high authorities in our own country, recognizing a distinct disease, under the latter title, or enteric fever, possessing most of the symptoms which mark typhus, but distinguished chiefly by a violent affection of the intestinal canal, producing diarrhœa during life, and revealing to anatomical investigation, a remarkable lesion of the mesenteric glands, and the glands of Peyer—while the English writers generally consider enteric fever as only a form of typhus, and not possessing those peculiar characteristics, that would entitle it to rank as a distinct disease. This latter opinion has been well stated by Cruveilhier, who in this respect differs from the majority of French physicians,—he says:—

"The miasma (from which he supposes the disease to originate) is carried by the air into the lungs, whose mucous membrane, so eminently absorbent, serves the purpose much oftener of absorbing, than of eliminating miasma, and other morbid causes, being, in this respect, in opposition to the skin and the intestinal mucous membrane. When the miasma is absorbed, it is carried into the current of the circulation, and infects the blood. Sometimes it acts immediately, at other times it needs, like a germ, a certain period of incubation. At the moment of explosion, a chill, more or less violent, takes place—a sort of centripetal movement, which announces that the economy is under the dominion of a cause which oppresses it, and that the digestive system is violently affected. Individuals have been known to die during the chill. The centrifugal movement, the reaction, heat, the developement of the pulse, follow. It is then that the miasmatic cause is carried to this or that organ—sometimes to the isolated or aggregated mucous follicles, when follicular enteritis follows; sometimes to the mucous membrane of the large intestine, when diarrhœa or dysentery supervenes. In other instances the miasmatic cause is directed to the lungs, or the brain—sometimes it is exhausted on a single organ, and again it is carried simultaneously, or successively, to several organs—in other words, in certain constitutions, the miasmatic cause reaches the large intestines, in others, the aggregated or isolated glands of the small intestine, in others the lungs, in others again the brain, &c., and these individual peculiarities, the various influences of all sorts, in the midst of which typhus is developed, explain the innumerable differences, which successive epidemics present." This description, as will be presently seen, applies accurately to the epidemic of 1847, where the same morbific causes produced, in different individuals, the diverse phenomena described. It may also be stated, in general terms, before proceeding to a more detailed description, that this disease was subject to the general laws of fever, viz, muscular debility, action and reaction, and the daily exacerbation impressed on all febrile action by the rhythmical movements of life; and exhibited the characteristic marks of typhus-stupor, dark color of the face, the eruption, peculiar odor, dark sordes, &c.

In seeking the causes of this epidemic, we are unable to find any which would indicate an essential difference between it and many preceding ones. Ireland, from which the imigrants chiefly came, has often suffered from sickness produced by general destitution. In 1847 an epidemic prevailed, caused by the wet changeable weather, which ruined the harvests, and reduced the people to a scanty supply of unwholesome food. Many a "Famine Fever," has stamped its dismal image on the minds of the older inhabitants. But so fearful a state of universal destitution and sickness, as prevailed in 1847, has not been recorded in the annals of that unhappy country. It seemed to be a summing up of the horrors of all former suffering.

To escape the terrible evils of famine and pestilence, which threatened them at home, the lower classes of the inhabitants hastened in crowds to take refuge in America. The ill ventilated steerages of the emigrant ships were filled to overflowing, the air becoming constantly more impure from the bad habits of the passengers; their stores of poor provisions were frequently insufficient to sustain them, the water was often bad, and limited in quantity—here, deprived of all excitement, without employment or exercise, their minds had time to brood over the fearful scenes they had left—fear, sorrow, anxiety, joined with the physical evils of their condition, tended to depress the vital energy, and the seeds of disease sown in their constitutions were thus nourished into life; the disease broke out with great violence, and many died before reaching land.

The following account of the phenomena of the fever, is derived from personal observations, made during my residence in the Blockley Hospital, Philadelphia, and the method of treatment discussed, is that pursued in that institution:—

As the patients arrived in every stage of the disorder, and were not able to give an intelligent account of their previous sufferings, and as few of the inmates of the hospital contracted the disease, sufficient data was not collected to determine the period of incubation. The cases of acute disease, ordinarily, do not produce action immediately upon application, but so many circumstances may affect individual instances, that a wide experience, only, will determine the general law. In ordinary typhus, from one to two weeks are supposed to elapse, before the effects of exposure appear.

The method of attack varied greatly. In some it commenced suddenly with a violent chill and headache, prostrating the patient instantaneously; but more frequently there were premonitory symptoms for several days,—such as more or less headache, accompanied by pain in the back, and general uneasiness in the whole system, an indisposition to all mental and bodily effort, alternate chilliness and flushes of heat, a shrinking tenderness of the whole body, loss of appetite—these, and corresponding symptoms, after lasting for two or three days, entirely prostrated the energy of the patient. Nausea or vomiting commonly attended the commencement of the disease. A chill of varying intensity marked the first stage, accompanied by a feeble pulse, and other signs of depressed vital action; febrile reaction, usually followed in a few hours, marked by a frequent pulse, often full and somewhat resisting in subjects who possessed much constitutional vigor; the skin was hot and dry, and the tongue furred; the symptoms of excitement continued to increase for several days, the pulse became more frequent and less forcible, mounting to 120, and sometimes 140 beats in a minute, the respiration was quick, the skin hot, dry and harsh, the tongue covered with a thick brownish yellow fur, the eyes heavy and bloodshot, the eyes of a dusky red color, the head aching, the brain oppressed, with stupor or slight delirium; an eruption appeared all over the body, particularly on the chest and abdomen, which lasted four or five days; these spots were small and close, resembling somewhat the eruption of measles in size and color,—occasionally in cases which terminated fatally, they lasted throughout the disease, changing to a purple hue as the fever assumed a more malignant character.

The following sketch which I made by the bedside of the patient, will serve as an illustration of the general termination of the fever when it proved fatal. The symptoms of nervous derangement which had existed ever since her entrance, increased; the skin still continued hot, but flabby, and covered with sweat; the eyes were bloodshot and nearly closed; the mouth half open, drawn on one side, the saliva trickling down; the teeth and gums were covered with hard sordes; the tongue dry, with black crusts, and incapable of protrusion; the breath of a peculiar and offensive smell; respiration labored, the chest heaving violently; the movement of the heart extremely feeble, the pulse almost extinct; the patient lay in a comatose state, with entire loss of voluntary power; medicine could no longer be swallowed, the head slipped down on the chest; there were now involuntary passages of a dark green color; the neck and other parts of the body were swollen and of a livid hue. Sometimes a furious delirium occurred in the last stage; and occasionally the vomiting of black sooty fluid, preceded death.

There were great differences in the state of the alimentary canal. It was always affected in the commencement of the disease; sometimes a diarrhœa continued throughout, with nausea or vomiting; again costiveness would alternate with looseness of the bowels, or would resist the operation of purgatives, throughout the disease. The cases exhibited every variety of condition in this respect. After death, the body frequently swelled, and became of a dark purplish hue, which appearances subsided after the lapse of a few hours.

The facts in relation to the post mortem appearances presented in Ship

Fever, were kindly furnished me by Dr. Knight, oue of the physicians of the institution.

Blood drawn during the different stages of the fever, rarely presented an inflammatory appearance; towards the fatal termination of the disease, it was dark, almost black, the coagulability of the fibrine being, in many cases, entirely lost.

Anatomical investigation proved the congestive nature of the disease. The brain and its membranes frequently showed marks of congestion, with occasional effusion of serum. Sometimes the signs of stagnation in the lungs explained the distressing dyspnœa that had existed during life. The liver, and particularly the spleen, were found to be softened and enlarged; this latter organ was invariably affected, its enormous distension giving it many times its natural weight, amounting in one instance, to over seven pounds; the softening of its substance gave no indication of inflammatory action, no coagulated fibrine was found in its cells, and few traces of inflammation appeared in the other viscera. Occasionally extensive lesions of the alimentary canal were observed. Sometimes the glands of Brunner were affected, and the isolated and aggregated glands of the ileum were found in a state of enlargement and ulceration.

Various plans of treatment have been adopted in typhus, according to the different theories which have prevailed in relation to the nature of the disease; thus, the stimulant, the antiphlogistic, the emetic plans have been each exclusively and eagerly advocated.

It is important to remember that successive epidemics of the same disease differ so greatly in their essential types, that a method of treatment generally successful in one, may be as fatal in another; this modification of disease proceeds from causes of which we are ignorant, and exerts a wide-spread influence, affecting not only the prevailing epidemic, but all other diseases existing at the same time; thus, when an epidemic has been marked by high, inflammatory symptoms, it has been found that other diseases not usually inflammatory, have for a time, assumed that type, and required an entire change of the ordinary treatment. An old writer, expressing this fact, says quaintly, "there are various constitutions of years, that owe their origins neither to heat, cold, dryness, nor moisture, but rather depend upon a certain inexplicable alteration in the bowels of the earth, whence the air becomes impregnated with such kinds of effluvia, as subject the human body to particular distempers, so long as that kind of constitution prevails." As no two epidemics of the same disease, present exactly the same form, so no two individual cases of the same epidemic, are exactly alike. Variety in unity, is a universal law, and no more in diseased than in healthy action, does Nature ever repeat herself. Each case, therefore, must be studied separately, and the medicines adapted to individual peculiarities. The general laws for treatment can be laid down, but the combinations required by the infinite variety of life, must be left to medical skill, exercised by the bedside of the patient.

It is evident, by the description already given, that the epidemic we are considering, did not present an inflammatory type—the symptoms presented during life—autopsical revelations—and a few experimental cases—all proved that venesection was not required, and could not be borne. The stage of high excitement which marked every case, contra-indicated the exclusive employment of stimulants. The most successful treatment, was one that adapted itself to the different stages of the fever, being sometimes a modified antiphlogistic plan, sometimes stimulating, and frequently simply expectant. Dr. Rush remarks, that "however excessive or deficient Nature may be in her attempts to throw off febrile disease, she rarely errs in pointing out the manner or emunctory, in, or through which, it ought to be discharged." The symptoms of a disease, therefore, furnish valuable indications of the method which Nature is taking to remedy the injury received, and her plan is frequently the best; but Nature is blind; her efforts must be watched, and sometimes guided; sometimes restrained; an effort salutary at the commencement, may be carried beyond the necessary point, by the habit or inertia of vital movement, which is as dangerous in disease, as it is essential to health; or a local disease, a symptom, which would constitute an important crisis of the general disease, if carried to the skin, becomes injurious, when directed to an organ essential to life; this not unfrequently happens. The skill of the physician is therefore indispensable to judge and direct the efforts of Nature; thus in the early stage of the fever, when nausea or vomiting, or a tendency to diarrhœa existed, an emetic, followed by some mild purgatives, sulphate of magnesia or castor oil, produced the happiest effects, frequently cutting the disease short at its commencement.

The majority of patients, however, arrived after the febrile movement was fully established; if the pulse was tolerably firm, and no signs of exhaustion present, the refrigerant diaphoretics answered the chief indications—the neutral mixture, combined with small doses of tartar emetic, the acetate of ammonia, or sweet spirits of nitre. To quiet nervous symptoms and wakefulness at night, Hoffman's anodyne, and a Dover's powder in the evening, were useful; the heat and dryness of the skin were relieved by frequent sponging with cold water. If headache existed, cold water or ice was applied to the head. The state of the bowels was of great importance; when costiveness existed, frequent purgatives were necessary, adapted to the condition of the system—in the early stages, when the pulse had considerable strength, the saline purgatives were most suitable; at a later period, rhubarb, alone or combined with calomel, or enemata of castor oil, or oil of turpentine, were preferred. When, on the contrary, there were distressing nausea or vomiting, or exhausing diarrhœa, other remedies were called for—an effervescing draught, a mustard plaster, or cups to the pit of the stomach, small pieces of ice continually swallowed, relieved the gastric symptoms; the chalk mixture, with kino or catechu, small doses of opium and ipecacuanha, with the addition of acetate of lead, rhatany, or some other suitable astringent, checked the excessive action of the bowels. Ipecacuanha alone or in combination with opium, proved a valuable remedy in checked and vitiated secretion.

When the disease held its course unchecked by medicine, stimulants became essential to the successful treatment, and patients arriving at the hospital in the last stages of collapse, were restored to life by their energetic exhibition. The skill of the practitioner was nowhere more strikingly displayed, than in the anticipation of this period of collapse; by seizing the first moment when the vital energy began to abate, although, to the inexperienced observer, the symptoms of excitement still existed in full force. The carbonate of ammonia, wine whey, and brandy punch, were the stimulants most commonly used; the former was given in emulsion, to the amount frequently of ʒi a day. The latter, one part of wine or brandy to two of milk, were taken, a table spoonful every hour or half hour.

Where the symptoms were in the last degree urgent, more powerful stimulation was necessary; pure brandy was frequently administered; mustard or cayenne pepper was applied to the extremities and the inside of the thighs; hot fomentations, blisters applied for their rubefacient effect only; the temperature of the skin being maintained by hot water, bricks or sand. These powerful applications were of course only called for in cases of extreme danger. Very frequently a state of great debility existed, when the excitement of fever had in a great measure passed away, the pulse being extremely feeble, though sometimes frequent, and the skin partially hot, and it was then essential that tonics, nutritive, and even stimulating food should be employed; but their effects were carefully watched, and their use discontinued when they tended to reproduce the febrile symptoms. The preparations of bark were often highly useful; opium in small quantities, repeated so as to produce a steady excitement; porter also was serviceable; nutritive food was often required at this time, the essence of beef or mutton, a table spoonful every hour, egg-nogg, and similar articles.

Great attention was paid to the thorough ventilation of the wards; perfect cleanliness enforced in the person and clothes of the patient, and the beds frequently changed.

Under this system of treatment, as pursued at Blockley, the mortality was about ten per cent., and during a part of the epidemic of 1847 it was as low as seven per cent.

In convalescence from typhus, the patient should be guarded with peculiar care. From the nature of the disease, an unusual relaxation of the whole system exists, and consequently a greater disposition to relapse from slight causes than in ordinary fevers. It is an important observation of modern pathologists, that when the general febrile symptoms have greatly diminished, the condition of the local malady by no means corresponds; that in the enteric form of typhus, the complete cessation of fever coincides with the time when the intestinal alteration becomes stationary. Experience has shown, that after six weeks, or even two months of apparent convalescence, a slight imprudence has renewed the entire inflammation, and diarrhæa, dysentery, or perforation and death have ensued. The appetite is generally keen at this time, but its demands must never be taken as a guide in the important matter of diet; the lightest aliments should be given during the first week of convalescence; gruel, chicken broth, well boiled potatoes, and, as strength increases, beef tea, roasted apples, the pulp of some kinds of fresh fruit, and the most digestible animal food, form a suitable regimen for the convalescent state.

Cleanliness, ventilation, and judicious exercise, are of the utmost importance. Dr. Johnson makes the following striking remark:—"Sick men recover health sooner and better, in barns, huts, and sheds, exposed occasionally to wind, and sometimes to rain, than in the most superb hospitals of Europe; pure air is, in this respect, alone, superior to all forms of care, and to all other remedies without such aid. When ground is gained by moderate diet, small in quantity, but stimulative of the powers of digestion, by exercises light but varied, and often repeated, it is effectually secured by the practise of washing the body in cold water, while the impressions of the active movements from the exercises and amuuements, are yet in force. The application of cold water in such condition has a powerfnl effect. It produces a salutary action, and if followed by friction, and the refreshment arising from clean apparel, it gives and confirms a mode, which may be called the mode or tone of health."

The importance of the hygienic means here referred to, cannot be too strongly insisted upon. It is the duty of the physician to modify them in their application to individual cases, but the employment of similar means, both physical and mental, for the restoration of health, forms an important part of the treatment of disease.

The liability to relapse, is observed to vary in different epidemics of the same disease. Comparatively few cases occurred in the epidemic of '47. The secondary fever which accompanies the relapse, is sometimes complicated with erysipelas, diarrhœa, or dysentery. The most frequent causes of relapse, are errors in diet, over exertion, or neglected bowels. In directing the treatment during relapse, the exhausted condition of the patient must always be borne in mind, and the activity of ordinary treatment greatly modified. The nervous system is highly susceptible at this time, and it is found extremely advantageous to administer an anodyne, when purgative medicines are employed, particularly where signs of intestinal irritation exist.

We have thus seen that the Ship Fever epidemic presented nothing peculiar or mysterious in its history. As it sprang from the same causes, so it presented the same phenomena, as the ordinary typhus, which always exists, in a more or less degree, in the crowded alleys and dismal cellars of our large cities. When the laws of health are generally understood and practised:—when a social providence is extended over all ranks of the community, and the different nations of the earth interlinked in true brotherhood—then we may hope to see these physical evils disappear, with all the moral evils which correspond to, and are constantly associated with them.

